# Massive sacrococcygeal teratoma in a preterm infant

**DOI:** 10.1002/ccr3.3722

**Published:** 2021-02-13

**Authors:** Pedro Maneira‐Sousa, Gustavo Rocha, Paulo Soares, Vanessa Arnet, Susana Guimarães, Ana Catarina Fragoso, Carla Ramalho, Estevão Costa, Hercília Guimarães

**Affiliations:** ^1^ Department of Neonatology Centro Hospitalar Universitário de São João Porto Portugal; ^2^ Faculty of Medicine University of Porto Porto Portugal; ^3^ Pathology Department Centro Hospitalar Universitário de São João Porto Portugal; ^4^ Department of Pediatric Surgery Centro Hospitalar de São João Porto Portugal; ^5^ Center of Prenatal Diagnosis Department of Obstetrics Centro Hospitalar Universitário de São João Porto Portugal; ^6^ i3S ‐ Instituto de Investigação e Inovação em Saúde University of Porto Porto Portugal

**Keywords:** intensive care, neonatology, preterm, sacrococcygeal teratoma

## Abstract

In extreme preterm infants, massive congenital sacrococcygeal teratomas with great hemodynamic commitment may be a situation for limitation of care.

## Graphical abstract

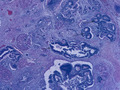

## 1 INTRODUCTION

A female infant of 26 weeks of gestational age with a massive sacrococcygeal teratoma needed active resuscitation after birth. She presented a rapid worsening course with hypovolemic shock, acidosis, and death. This case highlights the hemodynamic effects of large congenital teratomas with vascular components as a major cause of death.

Congenital sacrococcygeal teratomas (SCT) are germinal cell neoplasms derived from the misdifferentiation of the primitive streak during embryonic development.^1^ They are the most common neoplasm in neonates, mostly affecting females^1^, ^2^, ^3^, ^4^, ^5^ and are associated with numerous other congenital malformations.^5^ Preterm labor has a high prevalence among these patients, most probably related to the mass effect of the tumor.^1^ The perinatal mortality rate, although very inconsistent in the literature, may range between 13% and 50%.^2^, ^4^ Numerous factors have been described to predict a poor prognosis: tumor diagnosis before 20 weeks of gestation, changes in the placenta's morphology and size, intrapelvic extension of neoplasm, polyhydramnios, hydrops, low birth weight, low Apgar score, histologic characteristics of the neoplasm, preterm delivery before 30 weeks, and, more recently, neoplasm's volume and its vascular and/or solid component's proportion.^3^, ^4^ Cardiac failure and/or hemorrhage are considered the main causes of death.^2^ We report on the case of a preterm infant with a massive SCT with a high vascular component.

## 2 CASE REPORT

A female preterm infant was born to a 26‐year‐old healthy primigravida mother and a 30‐year‐old nonconsanguineous father. The first trimester was of low risk, and pregnancy was uneventful until 19 weeks, when the obstetric ultrasound revealed a sacrococcygeal mass with internal and external component (with 34 × 28 mm) suggestive of teratoma, why she was referred to our center. The amniocentesis showed a normal array comparative genomic hybridization (aCGH), and the echocardiogram was normal. An MRI was performed at 22 weeks gestation, which revealed a sacrococcygeal mass (60 × 70 × 57 mm) with mixt characteristics, predominantly solid, with the biggest cyst measuring 40mm, without invasion or compression of the pelvic structures or coccyx, Altman type 2. The subsequent ultrasounds revealed a fast growth rate of the tumor (80 × 60 mm) associated with profuse vascularization. The pediatric surgery team was informed. Until delivery, there were no other incidents.

Labor was spontaneous, and she was delivered vaginally at 26 weeks and 6 days of gestation, after a full cycle of antenatal steroids. The presentation was cephalic, and the birth weight was 1300 g. The Apgar score was 0, 0, and 5 at 1, 5, and 10 minutes, respectively. The parents expressed a desire for investment to be made in the child. For resuscitation, she needed ventilation with positive pressure, oxygen (maximum FiO_2_ 1.00), and intravenous adrenaline, with recovery of cardiac frequency after 6 minutes of resuscitation maneuvers. A single dose of surfactant was administered at the first hour of life.

**FIGURE 1:**
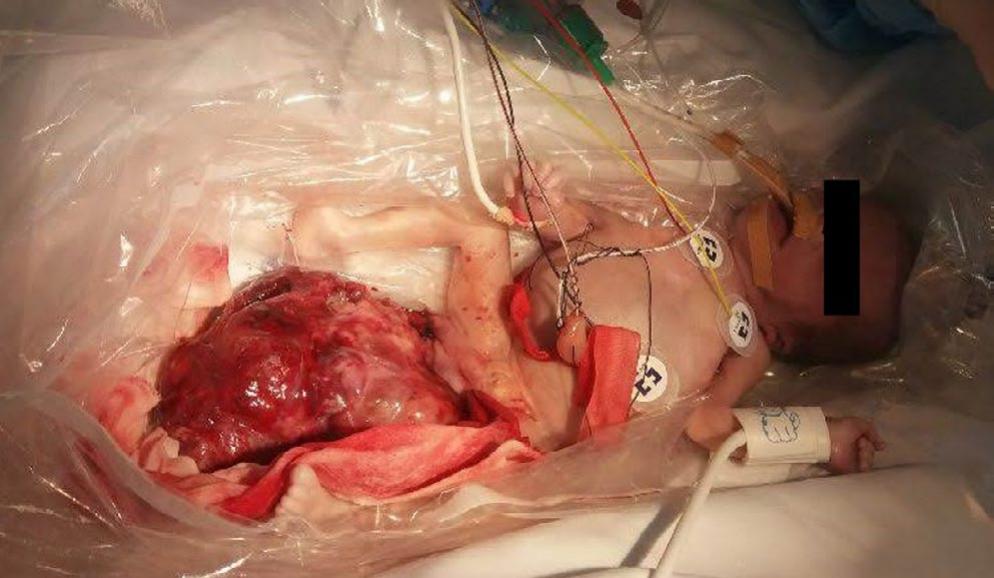
The preterm infant with hypovolemic shock due to a congenital massive bleeding sacrococcygeal teratoma

Right after birth resuscitation, she was admitted at the neonatal intensive care unit with signs of hypovolemic shock such as cardiac frequency 103 min^‐1^, respiratory rate 70 min^‐1^, systolic/diastolic arterial pressure 19/15 mm Hg ‐ mean 17 mm Hg, axillar temperature 34.1ºC and ventilated in synchronized intermittent positive pressure ventilation mode with volume guarantee (maximum settings: PIP 25 cmH_2_O, PEEP 5.0 cmH_2_O, Frequency 70 min^‐1^, FiO_2_ 1.00, VG 6.0 mL/kg). She was pale with increased capillary filling time and was presented with a reddish mass at the sacrococcygeal area measuring 14 × 7 × 4 cm, 750 g of estimated weight, with visible active bleeding (Figure 1). There were no other major clinical findings.

The patient, then, received substantial doses of adrenaline and was volumized several times with saline and glucose solutions, followed by transfusion of red blood cells (up to 30 mL/Kg), fresh‐frozen plasma (10 mL/Kg), and platelets (10 mL/Kg). Despite the efforts, the post‐transfusion hemoglobin level remained low, 5.8 g/dL. Also, she started ampicillin, gentamicin, and a perfusion of intravenous sodium bicarbonate, due to severe metabolic acidosis (pH 6.80, HCO_3_‐ 8.6, Base Excess −25.0). The cranial ultrasound revealed signs of subependimary hemorrhage and periventricular hyperecogenicity.

**FIGURE 2:**
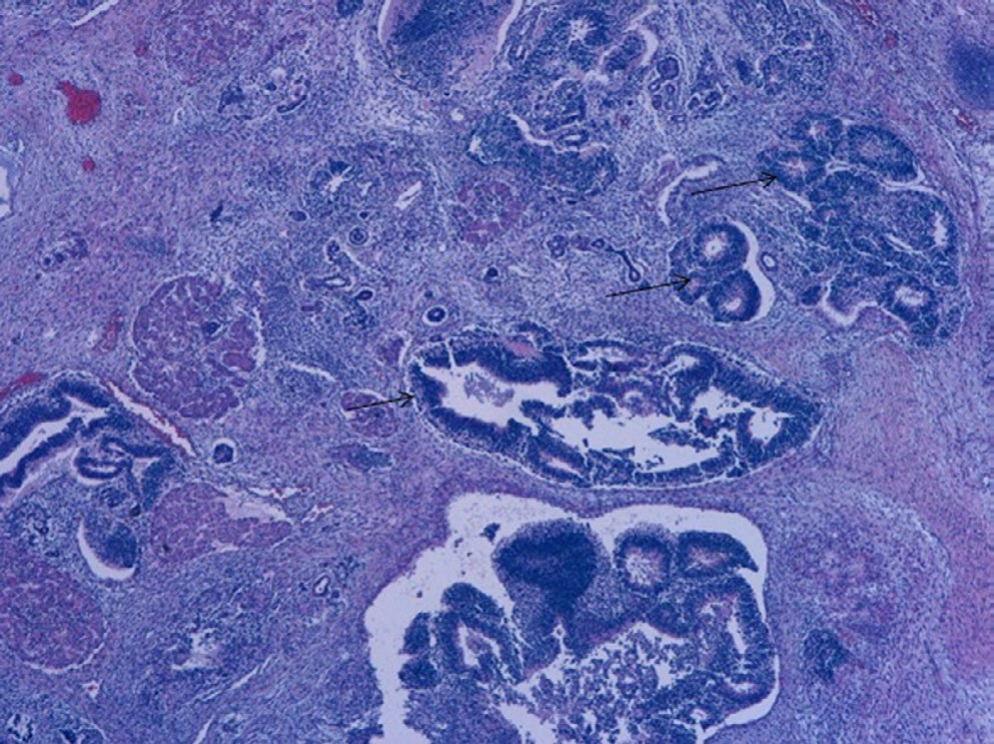
H&E, ×40. The predominant element was immature neuroepithelium (arrows)

**FIGURE 3:**
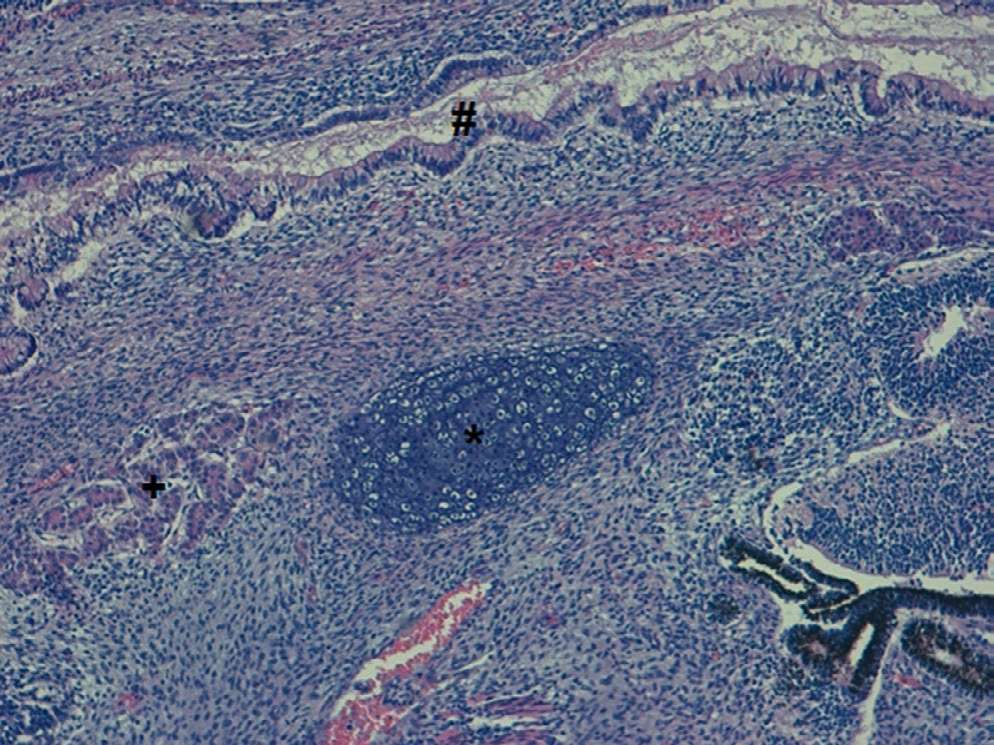
H&E, ×100 There were also mature elements: mesoderm derivatives (cartilage, *); ectoderm derivatives (retinal pigmented epithelium, arrow); endoderm derivatives (pancreatic tissue, + and intestinal epithelium, #)

So, accordingly to the clinical condition, she was submitted to an emergent sacrococcygeal patch by the pediatric surgery team. There was no time nor conditions to perform any surgical procedure or an embolization. However, despite the attempts to control the high‐output cardiac failure, there was a quick clinical worsening resulting in death after 6 hours of life, due to refractory shock and multiorgan failure in a fragile extreme preterm infant. The necropsy study confirmed the rich vascular nature of the tumor, with no other dysmorphisms, and the histologic examination confirmed the diagnosis of congenital immature teratoma, grade 3 (Figures 2 and 3). The placenta histology revealed signs of acute chorioamnionitis with mild chorionic vasculitis.

## 3 DISCUSSION

Sacrococcygeal teratomas often lead for a poor prognosis. Its embryologic findings show elements derived from the three blastodermal layers: ectoderm, mesoderm, and ectoderm. They can be classified in mature teratomas (fully differentiated tissues) which have a better prognosis and survival, immature teratomas (presence of variable proportions of primitive neuroectodermal tissue) and malignant teratomas (very uncommon in infants).^1^

As a consequence of the rapidly clinical worsening, the surgical options and adverse intraoperatory outcomes are scarce. Hence, patient characteristics, tumor morphology, and exsanguinating hemorrhage are important causes of early neonatal death.^2^

The differential diagnoses include myelomeningocele, lipoma, and the dermoid cyst, but also malignant tumors such as sarcoma of Ewing/PNET, neuroblastoma, and myosarcoma. The probability diagnosis is established on the basis of imaging detection of the tumor, ultrasound, and MRI, and in the finding of high levels tumor markers, such as alpha‐fetoprotein (AFP) and human gonadotrophic hormone (B‐HCG). High levels of AFP may indicate malignancy; unfortunately in the neonatal period, these values are usually high and only reach normal values about 9 months of age. The levels of these tumor markers were not evaluated in this reported case. The definitive diagnosis is established after anatomopathological analysis.^6^, ^7^, ^8^

In this case, we focus on two main mechanisms related for a bad prognosis for this tumor, the mass effect, and the rich vascular component. Although early preterm births themselves may not be the major cause of death in patients with SCTs, the large dimensions might be a likely cause of preterm delivery^4^ and severe hemorrhage during birth.^1^, ^2^ The chorioamnionitis could also be the cause of preterm delivery and will have played a negative role in the overall achievement of the patient. Also, an alternative delivery procedure—as cesarean section—could have reduced the risk of tumor compression and therefore bleeding during and after birth.^1^, ^2^ Consequently, although we did not consider these factors as the primary contributors for the patient outcome, prenatal diagnosis and measures might be an essential tool for the decision of perinatal procedures in the future.

Likewise, and most importantly, the rich vascular component of the tumor—which can justify the severe anemia, hypovolemia, and high‐output cardiac failure^4^—suggests that an important vascular steal phenomenon might be present in this case. In fact, these hemodynamic changes are the ones associated with the highest mortality rates in literature.^2^, ^4^ The factors that contributed to the patient´s death were the extreme prematurity (patient factor), high‐output cardiac failure, and vascularity of the tumor which can be correlated with the tumor size (tumor factors), and the hemorrhage from the tumor following delivery.

High‐output cardiac failure is a less common form of heart failure and results from the heart's inability to provide sufficient blood for the body's demand, either secondary to diffuse arteriolar dilation or possible bypass of the arterioles and capillary beds that can occur in vascular tumors. The problem lies with an increase in the body's demand for perfusion that the heart is not able to provide, even with a normal cardiac function.^9^ The fragile clinical state prevented the patient from compensating for bleeding.

Finally, this adverse cardiovascular status prevented a safe and effective surgical resection, as the poor hemodynamic condition constitutes a great surgical risk.^2^ Unfortunately, the clinical status in this case delayed all imaging tests to assess the intrapelvic extent of the tumor and made all medical and surgical efforts futile, including a more conservative surgical approach by ligation of the feeding blood vessels followed by a complete tumor resection^2^, although a patch was still performed with no success.

Teratomas are the most common neoplasm in neonates, but not commonly found in literature. This report highlights a rare but extremely challenging presentation in neonatology and surgery. Such cases are important for clinicians to be aware of in order to appropriately counsel future families in the antenatal period. This might also allow for decisions about limitations of care in the immediate postnatal period to be made. In this particular case, the clinical state of the baby after delivery, the degree of resuscitation required, the biochemical parameters following resuscitation, in addition to the known poor prognostic patient and tumor factors, make the futility of treatment obvious in retrospect.

There is a great difficulty on making withdrawal of care decisions in high pressure environments, especially when parents want investment, but the extreme prematurity, the aggressive resuscitation, the dimensions of the tumor, and the precarious hemodynamic and general condition of the patient may be useful to help with this decision.

## 4 CONCLUSION

Summarizing, this case emphasizes that SCT’s dimensions and its vascular components are the major determining hemodynamic changes that are crucial for the prognosis of these patients. Accordingly, the prenatal diagnosis and precise classification of the neoplasm would help the proper counseling in the antenatal period and the approach after delivery. A multidisciplinary involvement during pregnancy, including pediatric surgery, and a multidisciplinary approach after birth is of the utmost importance in clinical situations such as that reported in this clinical case.

## CONFLICT OF INTEREST

The authors have no conflict of interest or any financial support to declare. This manuscript has not been submitted or published elsewhere. Data sharing is not applicable to this article as no new data were created or analyzed in this study. All authors consciously assure that, for this manuscript, all the previous statements correspond to the truth.

## AUTHOR CONTRIBUTIONS

PM‐S: designed the study case and wrote the draft of the manuscript. All other authors: have contributed to data collection and manuscript review. All authors: have approved the final version of the manuscript.
